# Sperm protein markers for Holstein bull fertility at National Artificial Insemination Centers in Indonesia

**DOI:** 10.14202/vetworld.2020.947-955

**Published:** 2020-05-20

**Authors:** Zulfi Nur Amrina Rosyada, Mokhamad Fakhrul Ulum, Ligaya I. T. A. Tumbelaka, Bambang Purwantara

**Affiliations:** 1 Study Program of Reproductive Biology, Graduate School, IPB University, Bogor, Indonesia; 2 Department of Veterinary Clinic, Reproduction and Pathology, Faculty of Veterinary Medicine, IPB University, Bogor, Indonesia

**Keywords:** biomarker, fertility, sperm proteins, bull, Holstein

## Abstract

**Background and Aim:**

Holstein cows and heifers are widely bred in Indonesia by artificial insemination (AI) to increase population and milk production. Sperm fertility is modulated by genetic factors, but the analysis of sperm quality is still based on macro- and microscopic characteristics. This study aimed to analyze both sperm quality and proteins of Holstein bulls at different fertility levels.

**Materials and Methods:**

The frozen semen samples were collected from the Indonesia National AI Center. They were classified based on the reproductive efficiency data and were grouped into high fertile (HF) and low fertile (LF). Sperm qualities were evaluated by microscopic evaluation. The Holstein sperm proteins were extracted using phenylmethanesulfonyl fluoride as a protease inhibitor and the benzidine detergent extraction method. Discontinuous sodium dodecyl sulfate-polyacrylamide gel electrophoresis (SDS-PAGE) was conducted to analyze the molecular weights (MWs) of the sperm proteins. The data obtained were analyzed by a t-test using the one-factor bull fertility level, and Spearman’s correlation analysis was used to identify the correlation between the sperm microscopic evaluation parameters and protein bands.

**Results:**

The sperm motility post-freeze thawing was not significantly different between the HF and LF (p>0.05). The HF level had a higher percentage of viability, intact plasma membrane integrity, and intact acrosomes than the LF (p<0.05). Five protein bands were found in the SDS-PAGE of sperm proteins of Holstein bulls with different concentrations. Sperm proteins with MWs of 17.51 kDa, 14.87 kDa, 33.71 kDa, and 41.97 kDa were abundant in the Holstein bulls with an HF level, while 55 kDa proteins were abundant in the LF level of Holstein bulls. The sperm of Holstein bulls in the HF level contained proteins of about 33.71 kDa that were not detected in the LF.

**Conclusion:**

The sperm protein with a molecular weight of 33.71 kDa was predicted to be a specific protein biomarker that influences bull fertility. Sperm fertilization abilities were also determined by the sperm proteins, the morphology of sperm acrosomes, and the quality of plasma membranes. This method can be used to select bulls with high fertility to increase the population of Holstein bulls.

## Introduction

A bull’s fertility is determined by the ability of the sperm to fertilize the ovum to create a new individual (zygote). The bull fertility level is very influential on the reproduction efficiency, which is essential for effective and efficient breeding [1]. The application of artificial insemination (AI) as a reproduction technology takes advantage of frozen semen from a first-rate bull species in which the sperm could still be alive during the process of cryopreservation and impact the success of insemination [2]. The quality of frozen semen used for the insemination is considered to be significantly associated with the percentage of conception. This because the semen from one bull can affect the fertility and productivity of thousands of cattle. Therefore, the process of choosing the first-rate bull with the best cryopreservation quality of semen is of great importance to ensure successful insemination [3].

Dairy cattle, both male and female, have different characteristics from other local cattle. The data on their morphological bodies are essential and valuable management and tools that are useful in future strategies. They must be appropriately utilized to improve the productivity and fertility performance of bulls, which are also measured based on a breeding soundness examination (BSE) in addition to dairy cattle in terms of increasing milk production. Therefore, the high priority of bull selection in the AI center can help exploit their traits for the future genetic improvement of dairy [4]. In Indonesia, Holstein cattle are mostly bred by AI to increase their population and milk production. Frozen Holstein bull used for this purpose passed the BSE selection process, phenotypic screening (physically checking the external reproduction organ), libido observation, and standardized sperm quality character analysis. The quality of semen will deteriorate during the cryopreservation process, which can decrease fertility. Thus, BSE is considered unable to interpret the actual level of the bull’s fertility [5]. Sperm cryopreservation also induces the degradation of the sperm membrane, which can trigger the destruction of important cellular components, such as DNA and proteins [6]. A specific protein in the semen can change its function and affect the fertility of sperm [7]. It is reported that several proteins in the sperm and seminal plasma could be used as indicators of sperm characters, quality, and fertility levels [7,8]. Detected proteins in the sperm are related to the bull’s fertility, such as a heparin-binding protein with a molecular weight (MW) of 31 kDa [9] and phospholipase A2 with an MW of 16 kDa [10]. IZUMO1, which has an MW of 45 kDa, is a protein that plays a role in helping the acrosome reaction [11].

Although some studies have identified some types of indicator proteins, a specific indicator in Holstein bulls has not been discovered until now. This research aimed to analyze the sperm proteins and sperm quality of post-freeze thawing samples in Holstein bulls with different fertility levels. Therefore, the acquired protein analysis can function as a protein indicator of specific fertility in Holstein bulls.

## Materials and Methods

### Ethical approval

This study is excerpted from the ethical clearance examination of the Animal Care and Use Committee because semen collection using artificial vagina does not affect the normal physiology of the animal. However, The research was carried out following standard operational procedure SNI ISO 9001: 2015 No. 824 100 16072 at the Lembang AI center and SNI ISO 9001: 2015 No. G.01-ID0139-VIII-2019 at Singosari AI center and supervised by a veterinarian from both institutions. Ethical guidance and approval were provided by Lembang and Singosari AI center ethical committee on the responsible conduct of the use of bull for semen collection.

### Experimental animals

The study was conducted from January to May 2019. The Holstein bulls were selected from two National AI centers in Lembang, Bandung, West Java, Indonesia, and Singosari, Malang, East Java, Indonesia, based on the field reproduction efficiency data. Then, the frozen semen from six Holstein bulls (2-9 years old) divided into two fertility levels was collected and used in this study. All animals were clinically healthy, selected bulls were treated with proper management, and the frozen semen production was standardized between both AI centers.

### Holstein bull classification based on the reproduction efficiency data

The Holstein bulls used in this study were classified into two groups with different fertility levels: High fertile (HF) and low fertile (LF). The HF bull was classified based on the evaluation of reproduction efficiency data, including the first service conception rate (%FSCR) [12,13]. The data used for this experiment were the combination of AI data and pregnancy check, which came from the data of the National Animal Health Information System (iSIKHNAS) year 2017-2018 in West Java. Besides, the data were tabulated and selected according to their validities. They were also selected based on the technical capacity of the AI technician to successfully bred the cows/heifers in the first AI service (conception rate). The obtained %FSCR value of each bull was tabulated, and the mean value was determined, along with its standard deviation. Bulls with an FSCR value less below “mean−1Sd” were classified into the LF group, while the HF group belongs to bulls with an FSCR percentage above the “mean+1Sd” [13].

### The collection and evaluation of post-thawing spermatozoa

The frozen semen of Holstein bulls were taken from the semen bank in Lembang, Bandung, West Java, Indonesia, and Singosari, Malang, East Java, Indonesia. The bull fertility selection found six bulls with the following ID codes, 313111, 316121, 30775, 31088, 31084, and 30158, which were already classified based on their fertility level. The frozen semen straw from each of the six bull’s ID codes was taken from three batches in different production years with three replications per batch. Therefore, the total number of frozen semen used were 54 straws. The batch codes of the frozen semen adjust the availability at the semen bank of the AI center. Frozen semen in straws was kept in a transport container containing liquid nitrogen at −196°C and was then brought to the laboratory of the reproductive rehabilitation unit in Bogor Indonesia for evaluation. Before evaluating, the frozen semen was thawed in a 37°C water bath for 30 s. The contents of each straw were placed into a micro-tube. The post-freeze thawing sperm was evaluated for motility, viability, and abnormalities [14]. Sperm plasma membrane integrity (PMI) was evaluated by referring to Samardžija *et al*. [15], and intact acrosome (IA) was determined, according to Didion *et al*. [16].

The evaluation of sperm motility was conducted to determine the amount of sperm moving progressively. Semen, as much as 20 ml, was dropped on an object glass and then covered with a cover glass. The sperm movement was observed under the microscope at 400×. The sperm motility percentage was rated from 0% to 100% in ten fields of view. The viability and abnormality evaluations were conducted using an eosin-nigrosin dye. The semen was mixed with the eosin-nigrosin dye at a ratio of 1:3. The mixture was then made into a screw preparation on an object glass and was dried on a heating table for about 10 s. Both live and dead sperm were counted from ten fields of view. The live sperm will have a transparent color, whereas the dead sperm will appear red. The PMI of spermatozoa was tested using a hypo-osmotic swelling (HOS) test. HOS solution was made with a mix of 20-50 ml of semen and then mixed with 1 ml HOS solution to obtain a homogenous mixture. It was then incubated at 37°C for 30-45 min. Next, it was dropped on the object glass, covered with cover glass, and evaluated under the microscope. The spermatozoa, which have an intact plasma membrane, were indicated by a twisting tail or being inflated. Meanwhile, broken sperm were indicated by a straight tail. The minimal amount of sperm observed was 200 cells in ten fields of view.

Sperm IA evaluation was performed with two dyes. After thawing, semen was diluted with 0.9% of NaCl (1:5), was mixed with 0.2% trypan blue, and then was made into a screw preparation. The preparation was fixed by the mixture of 86 ml HCl 1N, 14 ml of 37% formaldehyde, and 0.2 g of neutral red solution for 2 min in a staining jar. The preparation was then rinsed with distilled water, dried, and stained with Giemsa dye (7.5%) for one night. Then, it was rinsed and dried again. The preparation was observed at a magnification of 40×, at which the sperm IA will appear red or dark purple on the anterior part, and the broken ones will appear white-greyish or have no color at all.

### The extraction and analysis of sperm proteins with sodium dodecyl sulfate-polyacrylamide gel electrophoresis (SDS-PAGE)

The frozen semen straw sample was thawed, put into a centrifuge tube, and rinsed three times. Rinsing was performed to remove the semen dilution substances by adding 1200 ml of PBS pH 7.4 to the sample, which was then centrifuged at 3000 rpm for 10 min to obtain the sperm pellet. The sperm proteins were then extracted by adjusting the suspension to 1 × 10^9^ sperms per 2.0 ml of buffer containing 62.5 mM Tris-HCl (pH 6.8), 2% SDS, 1.0 mM phenylmethanesulfonyl fluoride, and 23 mM benzidine as an inhibitor protease. The sperm was then vortexed for 10 min, followed by sonication [12]. The suspension was centrifuged at 900 rpm for 30 min at 4°C to obtain the supernatant, and cell debris was expelled from the cell. The aliquot of sperm protein extract was kept at −20°C to be analyzed by SDS-PAGE. The SDS-PAGE analysis was used to reveal the amount of proteins in the sperm and to determine their MW. The protocols used for SDS-PAGE analysis with silver staining dye were adopted from Laemmli [17]. The standard protein used in the SDS-PAGE process was a low MW marker (Spectra Multicolor Broad Range Protein Ladder, Fermentas Life Science) in which the MWs ranged from 10 to 260 kDa. The range of protein bands formed in the gel was measured and analyzed based on the range of protein bands compared to the running interval (retention factor [Rf]). The result of Rf analysis and weight log of marker protein bands were transformed into a linear regression equation. Then, the MW of each sperm protein band was obtained from the antilog of its MW. The intensity of the band thickness as an arbitrary unit was measured using ImageJ software (NIH, USA).

### Statistical analysis

The t-test analysis on a real level was 95% used in this research referring one factor called bulls fertility level, whereas the bull’s fertility level (high and low) was used as a treatment and the total number of bulls was considered repetitions. The correlations between the characteristic of sperm quality and fertility rate were analyzed using Spearman’s correlation test. The data were processed using Statistical Package for the Social Sciences program version 15.0 (IBM Inc., NY, USA).

## Results

### Fertility level classification of Holstein bulls

The Holstein bulls used in this research were classified into HF and LF groups based on the %FSCR using secondary data from the iSIKHNAS year 2017-2018 (Table-1). The data were tabulated and selected according to their validities. They were also selected based on the ability of AI technicians. The criteria used on the ability of AI technicians based on their success record to breed cows at the first services (conception rate), their experiences and certified profession as AI technician [16]. Table-1 shows that the group of cattle with AI <1000 and AI >1000 have a %FSCR value that indicates high fertility. The values were about 64.29%, 61.61%, and 63.11%, which are consistent with the usual range reported by Saha *et al*. [18] in which the standard conception value of the Holstein cattle was about 50%-77%. The %FSCR values of LF bulls were 21.15%, 22.22%, and 33.78%, which are under the normal range. One bull in the HF category with ID 312110 and one bull in the LF category with ID 30697 were not used in this research. This is because both of the males have been rejected, and the frozen semen straw stock was not available in the AI Center.

**Table-1:** Classification of Holstein bulls based on the percentage of first service conception rate^a^.

Group	Bulls ID	Batch code (year product)	first service conception rate (%)	Fertility rate^b^	Production
AI <1000	316121	AQ (2018)	21.15	LF	Lembang
	30158	II (2010)	22.22	LF	Singosari
	30775	JJ (2011)	64.29	HF	Singosari
	312110	-	74.03	HF	Lembang*
AI >1000	31084	QQ (2018)	33.78	LF	Singosari
	30697	-	35.28	LF	Lembang*
	313111	AR (2019)	61.61	HF	Lembang
	31088	PP (2017)	63.11	HF	Singosari

a Source: iSIKHNAS data year 2017-2018 processed,

b LF=Low fertile, HF=High fertile,

* Not used in this research because the stock samples were not available, AI=Artificial insemination

### The quality of post-freeze thawing sperm of Holstein bulls and the correlation with fertility level

The evaluation of the post-freeze thawing sperm of Holstein bulls is on the two levels of fertility: High and low. There are some significant differences (p<0.05) in the sperm viability, abnormality, PMI, and IA for both high and low fertility. The spermatozoa motility parameter was not significantly different between HF and LF bulls. The sperm post-freeze thawing evaluation results by microscopy are shown in Table-2.

**Table-2:** Evaluation of Holstein bulls’ frozen semen post-thawing with different fertilities.

Parameter	straw (n)	High fertile	Low fertile	p-value
Sperm motility (%)	54	45.00±4.59	43.70±3.27	0.24^ns^
Sperm viability (%)	54	81.33±4.26	78.98±4.20	0.03
Sperm abnormalities (%)	54	6.75±3.72	10.16±3.97	0.02
Sperm PMI (%)	54	80.08±4.64	71.41±5.31	0.00
Sperm IA (%)	54	50.24±3.66	45.15±3.61	0.00

Significant p<0.05 difference; ns: Non-significant; PMI=Plasma membrane integrity, IA=Intact acrosome

The correlation of sperm quality with fertility level is shown in Table-3. The results showed that sperm motility was positively correlated (0.142) to fertility level, but this was not significant (Table-3). The level of fertility of Holstein bulls was significantly positively correlated with viability (0.303), PMI (0.721), and IA (0.864) and negatively correlated with sperm abnormalities (−0.466) (Table-3).

**Table-3:** Correlation between fertility rate and the parameter of Friesian Holstein bulls post-thawing.

Parameter	FR	Sperm motility	Sperm viability	Sperm abnormalities	Sperm PMI	Sperm IA
FR	1	0.142	**0.303**	−0.466*	0.721*	0.864*
Sperm motility		1	0.608*	−0.380*	0.039	0.218
Sperm viability			1	**−0.325**	**0.297**	0.384*
Sperm abnormalities				1	−0.439*	−0.576*
Sperm PMI					1	0.765*
Sperm IA						1

Significant: p<0.05 bold, p<0.01 bold

* , FR=Fertility rate, PMI=Plasma membrane integrity, IA=Intact acrosome

### The analysis of spermatozoon proteins with SDS-PAGE

**Figure-1:**
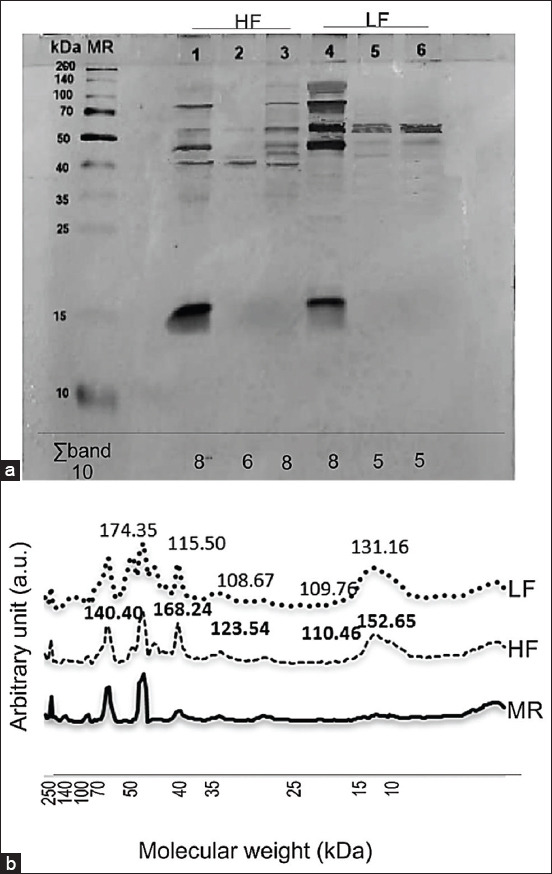
Protein profile of Holstein bull sperm with different levels of fertility (a) SDS-PAGE gel, (b) spectra of band intensity. HF=High fertile, LF=Low fertile, MR=Marker, a.u.=Arbitrary unit.

The sperm protein of Holstein bulls between the level of HF and LF spermatozoa contained different protein bands with various MWs (Figure-1). There was no difference in the amount of protein bands between HF and LF levels. The grey color density spectra resulting from protein band indicators between HF and LF were different, as shown in Figure-1.

**Figure-2:**
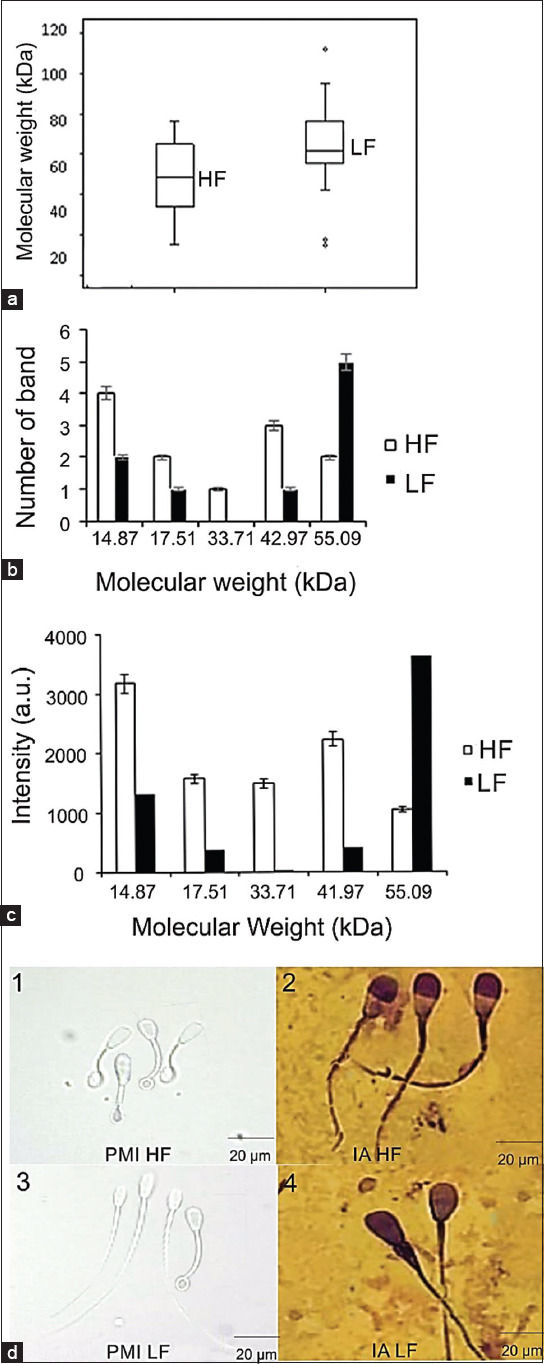
Number of sperm proteins and morphology of Holstein bull. (a) The distribution of protein molecular weights of Holstein bull’s sperm in the high fertile (HF) and low fertile (LF) level; (b) the sum of bands for each protein molecules of Holstein bull sperm with different levels of fertility; (c) intensity rate of each Holstein bull sperm proteins in the HF and LF level; (d1) sperm PMI of the HF; (d2) sperm intact acrosome (IA) of the HF; (d3) sperm PMI of the LF; (d4) sperm IA of the LF.

The average number of protein bands at the HF level was eight, and at the LF level, it was found to be five protein bands. Electrophoresis results also showed differences in the color thickness of protein bands. The bands had different intensities, which are shown by the peak of the spectra (Figure-2). Five protein bands with different MWs were predicted as marker proteins for fertility. These included proteins with MWs of 55 kDa, 41.97 kDa, 33.71 kDa, 17.51 kDa, and 14.87 kDa. The peak intensity of the arbitrary unit showed the difference in the concentration of spermatozoa protein at a specific MW. The peak intensity of the arbitrary unit with an MW of 55 kDa was shown to be at a higher LF level. The peak intensity of the arbitrary unit in HF was shown to be higher than LF on proteins with MWs of 41.97 kDa, 33.71 kDa, 17.51 kDa, and 14.87 kDa. The thicker band printed on the gel indicates a higher concentration of protein [19].

## Discussion

### The post-freeze thawing quality of Holstein bull sperm and the correlation with fertility level

For the post-thawing sperm motility, there was no difference between HF and LF bulls (p>0.05). The sperm motility percentage was categorized normal based on the SNI 4869-1:2017 requirement for post-thawing frozen semen with minimum mobility of 40% [20]. Karunakaran *et al*. [8] showed that generally after the process of freezing and thawing, sperm motility decreases by around 34%-46%. Cryopreservation and the freeze-thawing phase causes detrimental effects, such as stress induced by chemical, physical, and osmotic pressure, or changes in the temperature of the sperm [21]. Decreased motility is also caused by damage to mitochondria that affects the production of adenosine triphosphate (ATP), which is the energy resource required for sperm to be motile. Decreased sperm motility is caused by mitochondrial damage [22]. Sperm mitochondria are composed of matrix helices located in the middle part (mid-piece). The main function of mitochondria is to produce ATP through oxidative phosphorylation and provides energy for the sperm to be motile. Mitochondria dysfunction affecting either the structure or genome can interfere with sperm motility [23].

The sperm viability percentage of post-thawing at the HF level was higher than the LF level (p<0.05). The process of cryopreservation can also decrease viability. The process of freeze thawing can decrease sperm viability as much as 45% [24]. Antioxidants present in bovine semen substantially decreased and were not sufficient to protect sperm integrity against oxidative stress during cryopreservation. This makes sperm cryopreservation more susceptible to damage due to the production of reactive oxygen species [25]. The percentage of sperm post-freeze thawing viability can be considered good if the AI is around 64%-80% [26]. Therefore, the percentage of sperm post-freeze thawing viability for both fertility levels was in good condition to be used for AI.

In terms of post-thawing sperm abnormalities, there was a significant difference (p<0.05) between HF and LF bulls. The percentage of post-thawing abnormality for the HF level was lower than that for the LF level. Sperm is faced with physiological and structural challenges that are caused by changes in osmotic balance, oxidative stress, and formation of intracellular ice crystals, which can damage live sperm cells during the freezing process [25]. Changes in sperm morphology and mitochondrial damage are induced by cryopreservation [24]. Sperm abnormalities, especially head abnormalities, are generally considered to be uncompensated and possibly interfere with the fertility of bulls. The percentage of sperm abnormalities from dairy breeds was around 7.19% [27]. Since the percentage of post-freeze thawing sperm abnormalities is higher than LF, it will be one of the factors that can decrease the ability of the sperm to fertilize the oocyte.

The sperm PMI and IA for the HF level were higher than that for the LF level (p<0.05). The integrity of the plasma membrane is essential for frozen semen because it becomes a prerequisite that determined the ability of the sperm to survive. The sperm plasma membrane plays an important role in protecting cell organelles, especially the spermatozoa acrosome [28]. The spermatozoa acrosome has an inner acrosome membrane (IAM) and an outer acrosome membrane that will play a role in the release of oocyte penetration enzymes during the acrosome reaction at fertilization [29,30]. The integrity of the acrosome is the key to successful fertilization. Spermatozoa with IA are capable of penetrating the zona pellucida and fusing with oocyte plasma membranes [31]. Acrosome integrity is an essential indicator in assessing frozen spermatozoa fertility (Table-3). The highest sperm PMI value for the frozen semen of bulls is about 86.75% [32]. However, the percentage of sperm IA of bulls with double stain is around 34%-39% [33]. A sperm acrosome intactness has a significant effect on FSCR and contributes to determining the fertility rate of the bull [34]. Damage to the plasma membrane and acrosome membrane of sperm will lower the potential of sperm fertility [35] (Table-3). The percentages of sperm PMI and IA in this research were normal.

Based on the evaluation, the parameter of frozen semen post-thawing that was performed showed that both levels of bull fertility yield a good result (Table-1). This is because the bulls used in this study were from the National AI Center, we selected bulls that were treated with good management, and the frozen semen production procedures had been standardized. Sperm needs high motility, normal morphology, good viability, and an IA to fertilize an oocyte [36].

The result of this research shows that sperm motility is positively correlated to the level of fertility, but was not significantly different between HF and LF clusters (Table-3). The fertility level of Holstein bulls is significantly positively correlated with sperm viability (p=0.303), PMI (p=0.721), and IA (p=0.864), but it has a negative correlation toward sperm abnormality (p=−0.466). The percentage of sperm viability, PMI, IA, and sperm abnormality has a significant role in determining the potential of sperm fertility. The correlation among these parameters is useful to determine the potential and fertility level of Holstein bull sperm to result in pregnancy. Viable sperm have an intact sperm PMI so that the cell organelles in the sperm, and the integrity of the acrosome can be protected [28]. Nutrients and ions for metabolism are also available. Breaking of the sperm plasma membrane can cause a leak of the intracellular contents that will affect ATP production so that the motility of sperm is shaken off [37].

### The analysis of sperm protein by SDS-PAGE

The average number of protein bands found in the HF level was eight. Meanwhile, the average number of protein bands of the LF level was five. Based on the SDS-PAGE results, five protein bands with different MWs were predicted as indicator proteins of fertility. The electrophoresis result also shows the different color thickness of the protein bands. This suggests that there is a correlation between the thickness of the protein band and the concentration of proteins. The thickness of the band reflects the different substance of protein concentration. Based on this study, the thickness of the arbitrary unit of each band described contains the protein concentration. According to Subagyo *et al*. [19], the thicker the band printed on the gel, the higher the concentration of protein.

The distribution of of sperm proteins MWs between HF and LF levels was different (Figure-2a). The distribution of MWs of sperm proteins for the HF level tended to be balanced; meanwhile, for the LF level, the distribution was mostly 60 kDa or above. Therefore, the distribution of MW of proteins might be related to the fertility rate.

Figure-2 shows that the number of bands with an MW around 14.87 kDa was higher in the HF level than in the LF level. The protein with an average MWs of 15 kDa is called A-Kinase Anchor Protein 82 (AKAP 82) [38]; meanwhile, the protein with a MW between 17 and 18 kDa is called AKAP 3 [39]. Both of these proteins are localized in the sperm’s tail and are known to regulate motility. The regulation of sperm motility occurs because AKAP82 and AKAP3 bind to cyclic adenosine monophosphate, a second messenger, which then activates protein kinase A (PKA). PKA in spermatozoa tails interacts with tyrosine-phosphorylated AKAP during capacitation to increase the motility of spermatozoa [40].

Proteins with a MW of 33.71 kDa are expressed more in protein bands with HF levels than LF levels when viewed from the peak intensity of the arbitrary unit (Figure-1b). Phospholipase C-zeta (PLCz) protein is synthesized as a spermatozoa protein with MWs in the range of 33-34 kDa. PLCz plays a role in triggering the occurrence of calcium oscillation and oocyte activation after spermatozoa are in the female reproductive tract, which is very influential on the success of fertilization in mammals [41]. The enzymatic protein PLCz is attached to the plasma membrane part of the spermatozoa and partly to the internal membrane of the acrosome [42]. PLCz increases the occurrence of cytoplasmic calcium oscillations that are suitable for triggering fusion in several mammalian species [43].

Based on the results, a protein with an average MW of about 41.97 kDa was highly abundant in the HF level. Meanwhile, a protein with an MW of 55.08 kDa was abundant in the LF level. The protein in sperm with an MW of 42 kDa is tyrosine-phosphorylated SPACA1 (tp-SPACA1), which is expressed during the process of sperm maturation in cauda epididymis. The function of tp-SPACA1 is to protect the acrosome and fertility of sperm [11]. In addition, if it undergoes some shortage, it can be a sub fertile [44]. tp-SPACA1 is the main phosphorylated tyrosine protein found in the sperm head in the equatorial segment area of the cow, pig, and sheep spermatozoa. The equatorial segment contains tp-SPACA1, which functions to initiate fusion competence in the plasma membrane after an acrosome reaction [45]. This shows that sperm IAMs at the HF level are in better condition than at the LF level (Table-2). Therefore, tp-SPACA1 works well at the HF level.

Proteins with MWs ranging from 55 to 60 kDa were abundantly found in the LF level. Zahn *et al*. [46] stated that the protein with a MW of about 53-55 kDa is proacrosin. Gurupriya *et al*. [47] stated that proacrosin is found in the acrosome. When the sperm acrosome is damaged, or there is early capacitation at the time of freeze-thawing, it can stimulate the premature transformation of proacrosin into the enzymatic protein called acrosin. Kaiin and Gunawan [48] reported that capacitated sperm cause the number of sperm undergoing an acrosome reaction to increasing. Based on the sperm IA evaluation results (Table-2), it is revealed that there is a connection between the intactness of the acrosome’s veil and the density level of protein that is predicted to be proacrosin according to its MW. Based on the individual data of Holstein bulls with the IDs 316121, 31084, and 30158 that were classified into the LF level, it has a low intactness level of the acrosome’s cap.

## Conclusion

The sperm protein with an MW of about 33.71 kDa is predicted as a sperm protein biomarker in the Holstein bulls. It was predicted as a specific protein biomarker that influences the bull’s fertility. The presence of specific sperm proteins, the morphology of the sperm acrosome, and PMI could be significant determiners of sperm fertilization abilities.

## Authors’ Contributions

BP brought the general concept of the study and supervised the management of the experiment. ZNAR, MFU, LITAT designed a detailed part of the experiment. ZNAR conducted the field survey, collected samples, and examined them in the laboratory, MFU contributed to the statistical analysis. EH and TH provided frozen semen and fertility data. ZNAR, MFU and LITAT, drafted and revised the manuscript. BP and ZNAR finalized the manuscript. All authors have read and approved the final manuscript.
